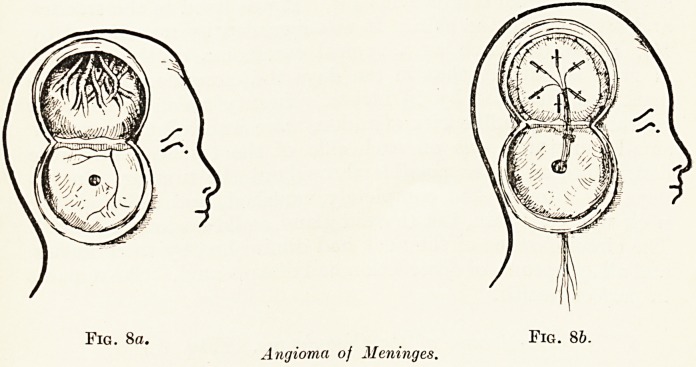# Radium and Its Surgical Applications
*From a Paper read at a Meeting of the Bristol Medico-Chirurgical Society held at the University of Bristol on 12th February, 1929.


**Published:** 1929

**Authors:** H. S. Souttar

**Affiliations:** Surgeon, London Hospital


### RADIUM AND ITS SURGICAL APPLICATIONS.*

BY

H. S. Souttar, C.B.E., F.R.C.S.,
*Surgeon, London Hospital.*

---

The discovery of radium by Madame Curie in 1898 opened up a field of physical research the limits of which it is impossible to see. Although it was found at an early stage that radium had a definite action upon living tissues, this appeared to be purely destructive, and it is only recently that the selective action of radium upon cancer cells has been adequately demonstrated. Depending as it does upon $\gamma$ rays, this selective action involves the use of screens to cut out those rays which are purely destructive, and the success of modern methods has depended very largely on accurate methods of screening. So great has been this success, that it marks a new epoch in the treatment of malignant disease, and it would seem that its extension to the general treatment of cancer is only a matter of discovering suitable methods of application.

The most remarkable property of radium is the fact that it steadily decays, and passes through a series of gradations, some of them far more active than radium itself, ultimately becoming inert lead as the final product of its disintegration. Radium itself stands half-way in the series of disintegration products,

* From a Paper read at a Meeting of the Bristol Medico-Chirurgical Society held at the University of Bristol on 12th February, 1929.

descending from uranium and finishing as lead. Taking as representative of each product the period required for half the element to disappear, the "half-value period" varies between five thousand million years for uranium, 1,750 years for radium and three minutes for radium A. For radon, the immediate and by far the most important product of radium, the "half-value period" is 3·85 days, and it is to the rapid decay of this product that we owe the surgical possibilities of radium. The radiation thrown out by radon as it disintegrates has the extraordinary power of selective destruction of cancer cells.

To understand the meaning of "decay" it is necessary to grasp the modern conception of the atom as a minute solar system in which the nucleus is the sun, whilst the electrons whirling round it are the planets. The nuclei are complex structures built up of protons and electrons, each carrying respectively an exactly equal positive and negative charge, whilst the planetary electrons, each carrying the same negative charge, are identical with $\beta$ particles.

The simplest atom of all is the hydrogen atom, consisting of a single proton as nucleus, with a single electron flying round it. Next in order comes the helium atom, its nucleus built up of four protons and two electrons welded together, whilst its planetary system consists of two electrons whirling round the nucleus. To us its importance can scarcely be exaggerated, for the nucleus is identical with the $\alpha$ particle, so that an $\alpha$ particle may be regarded as a helium atom deprived of its two planetary electrons, whilst it does not require much imagination to see that it must carry a positive charge exactly double the negative charge carried by each electron. When we point out that the electron, set free to fly through space, is the $\beta$ particle, and that with its liberation is bound up the production of $\gamma$ rays, it will be realized that the helium atom carries in miniature the whole physical basis of our subject.

Contrasted with these simple structures, the radium atom presents an enormous complexity. Its nucleus is a dense cluster of 226 protons and 138 electrons, some at least of which are grouped together to form $\alpha$ particles, whilst around this nucleus whirl no fewer than 88 electrons. The formation is unstable, and $\alpha$ particles are being constantly discharged from the nucleus, $\beta$ particles from the nucleus and from the planetary system. As each particle is projected into space the characters of the atom are changed. It is no longer radium but one of its derivatives, and this is the physical interpretation of "decay." Radium becomes radon, radon becomes radium "A," which is so unstable that within a few minutes it has lost an $\alpha$ particle and become radium "B." Finally, stability is reached in an atom indistinguishable from that of lead, and so far as we know no further change occurs.

One is apt, however, from such a description to gain an entirely false impression of the relative dimensions of the atom and its nucleus. The most extraordinary thing about this complex structure is the minute space occupied by the nucleus and the electrons, so minute indeed that our comparison with the solar system scarcely leaves enough room. Accurate measurements of the dimensions of the hydrogen atom show that it may be fairly represented by one golf ball circling round another in an orbit a mile across. On the same scale the diameter of a single tissue cell would be about one hundred thousand miles. When we talk about the impact of $\beta$ particles, it is well to bear these facts in mind, and to remember also that the tissue cells themselves are made up of atoms as empty as those we have described.

We are now in a position to understand the property of radium which gives to it for us its peculiar value, the radiation which accompanies decay. As we have seen, it takes three forms, the $\alpha$, $\beta$, and $\gamma$ rays, and we may now sum up briefly the very different characteristics of these three forms.

The $\alpha$ rays consist of particles identical with the nucleus of helium, each carrying a positive charge of electricity, and they are projected from the nucleus with a velocity of 10,000 miles a second. In spite of this terrific velocity, they have little power of penetration, and are stopped by a thin sheet of paper. Even in the air they rarely travel farther than a couple of inches, so that although they carry the great bulk of the energy produced by the dissolution of radium, they are of no practical value in surgery.

The $\beta$ rays are formed by electrons, of very small mass, carrying a unit negative charge of electricity, and projected with any velocity up to 180,000 miles a second, falling just short of the velocity of light. The soft rays of low velocity are easily stopped by a thin sheet of aluminium, but the hard rays of high velocity can pass through 0·2 mm. of silver. All but a small percentage of $\beta$ rays are, however, stopped by 0·3 mm. of platinum or by a centimetre of the body tissues. Their surgical action is therefore very local, and it is often better to avoid it altogether by the use of screens.

The $\gamma$ rays are ether waves of exceedingly short wave length, less than 1/5000 of that of light. They are set up apparently by the discharge of the $\beta$ particles, they travel with the velocity of light, and their power of penetration is so great that they are scarcely affected by the screens which almost abolish $\gamma$ rays, they can pass through several inches of lead, and they are only reduced by about 50 per cent. in passing through four inches of the body tissues.

It is thus upon the $\beta$ and the $\gamma$ rays that the surgical action of radium depends. The former are destructive in their effect, and they are chiefly used in surface applicators for the production of superficial scarring. The $\gamma$ rays, on the other hand, have two very remarkable effects: they stimulate the activity of the tissue cells, and especially the production of fibroblasts, and they have a definite selective action on tumour cells, probably at the moment of their division, causing them to disintegrate and disappear. Their total effect when they can be suitably applied is to cause the disappearance of a malignant tumour and its replacement by a scar.

We must say one word more about radon, the emanation of radium and the essential agent through which it acts. It is a heavy, inert gas with no chemical affinities, and it decays with such rapidity that in 3·85 days one-half of it has vanished, whilst at the end of 9 days only a fifth remains. So long as it remains in contact with radium the decay is made up by production, so that after a certain time equilibrium is established. If radium is left undisturbed this is reached in about a month, and thereafter production and decay exactly balance. At this stage radium has reached its maximum radio-active power, and the radio-active power of a milligramme of element is defined as a millicurie.

The fact that radon can be separated from its parent radium and packed into minute containers is of great practical value. So minute is its bulk that one thousand millicuries only occupy the space of 0·6 of a cubic *millimetre*, so that it can be put into capillaries of infinitesimal dimensions. Its action is identical at any moment with that of a certain amount of radium, but its power is constantly diminishing, so that in 3·85 days it is down to one-half and in 9 days to one-fifth. This, however, is easily neutralized by starting with a larger dose, and 1·5 millicuries of radon gives a total radiation equal to that of one milligramme of element left in for a period of eight days.

### APPARATUS AND MODES OF APPLICATION.

The apparatus used in the application of radium is of the simplest description, and, excluding the flat applicators which are chiefly used by dermatologists, it consists principally of needles containing radium element and seeds containing radon gas.

Needles are small platinum tubes varying in diameter from 1·5 to 3 millimetres, with a point tipped with iridium, and an eye through which a stout silk thread can be passed. A small cavity occupies the whole of the needle except the eye and the point, and this is packed with insoluble radium sulphate, the utmost care being taken to secure absolute uniformity in packing. The wall of the needle should be not less than 0·5 millimetres in thickness, so as to cut off the whole of the $\beta$ rays. The length of the needle will vary with the requirements of the case. At the London Hospital our needles are 2, 3, 4 and 6 centimetres in length, and we have the convenient arrangement that each needle contains one milligramme of radium per centimetre of length.

Seeds are minute capsules containing radon gas. Usually the gas is sealed up into a tiny glass capillary, which is then inserted into a tube of platinum, gold or silver, to cut off the $\beta$ radiation. Theoretically platinum is the most efficient screen, but personally I have found silver screens entirely satisfactory. Silver presents the advantages that it is cheap and easily worked, and I have recently elaborated a plan by which the radon is drawn directly into a long silver tube, which is then cut up into suitable lengths. The method of cutting ensures an absolute seal, and the saving of time and trouble, and of personal risk to the laboratory assistant, is considerable.

These needles and seeds provide small sources of $\gamma$ radiation which may be either introduced into the tissues or fitted into external applicators.

Introduction into the tissues is controlled by the empirical observation that the $\gamma$ radiation from a milligramme of radium can in a period of about five days destroy the cancer cells in a cubic centimetre of tissue. The total amount of radium required in milligrammes is thus the volume in cubic centimetres of the tissue to be irradiated, although in the case of large growths, from the effect of cross radiation, a smaller amount will suffice. It must be distributed evenly, the needles or seeds being placed about two centimetres apart, and it must be placed chiefly in the growing edge of the tumour. The needles are attached to stout silk threads, since they contain radium element and their loss would be disastrous. The seeds may be attached to fine silk threads and be removed later, or they may be simply inserted and left in the tissues, where under most circumstances they do no harm. The insertion of seeds is less likely to be followed by the slight sepsis which sometimes follows the insertion of needles, whilst they have the great advantage that the patient can go home. The action of the needles is, however, uniform and not diminishing, and on this account they are preferred by some surgeons.

The treatment of a case of carcinoma of the breast furnishes an excellent example of the introduction of radium needles. The diagram (Fig. 1) indicates the arrangement worked out by Mr. Keynes at St. Bartholomew's which has given brilliant results. Two groups of needles are inserted into and beneath the tumour itself on different planes, two rows of needles follow the lymphatics along the borders of the two pectoral muscles, a third group occupies the axilla, a fourth is inserted beneath the clavicle in the region of the costo-coracoid membrane, a fifth is placed above the clavicle, and a sixth group is inserted one in each of the upper five intercostal spaces and one in the rectus sheath. The actual number of the needles will vary between forty and fifty with the dimensions of the breast and of the tumour, and the total amount of radium between 75 and 100 milligrammes. The needles are left in place for from seven to nine days, and are then removed.

The tongue is an ideal site for the use of seeds (Figs. 2, *a*, *b*). About eight or twelve are required, each containing from 1 to 2 millicuries of radon gas, and they are inserted into the base of the growth, usually through the tongue itself. In this way the actual growing edge is subjected to an intense cross-fire radiation, whilst as access is obtained directly through the soft tissues of the tongue, the treatment of growths far back, in situations quite inaccessible to ordinary surgery, is perfectly simple. In most cases the tumour will disappear by magic, and three weeks later it will have been replaced by a contracting scar. The glands on both sides of the neck are now dissected out in the most thorough manner, and three weeks later the treatment is complete by the external application of radium needles mounted on a thick wax collar made of Columbia paste.

For the irradiation of tumours or of glandular

FIG. 1.—*Carcinoma of Breast.*

FIG. 2*a*. *Carcinoma of Tongue.* FIG. 2*b*.

FIG. 3.—*Carcinoma of Palate.*

deposits by external application it is essential that the radium should be supported at some distance from the skin, as otherwise certain areas of the skin will receive a dose which they cannot tolerate and will break down. A convenient plan is to mould a sheet of Columbia paste —a mixture of beeswax 100, paraffin wax 100, and pine sawdust 200—to the region. The wax should be 1·5 centimetres thick, and on its outer surface radium needles should be planted, screened in the ordinary way with 0·5 millimetres of platinum. Small needles containing from 0·5 to 1·5 milligrammes of radium should be distributed at a distance of 1 centimetre apart over the surface, whilst special regions, such as the eye or nose, can be protected by the introduction of thin lead plates into the wax mould. The whole appliance should be covered with a thin sheet of lead for the protection of the nursing staff. Even more convenient than wax in many cases is a sheet of spongy rubber; it has the great advantages of lightness and adaptability, and the needles are easily attached to it by strapping. The period of application varies with the strength of the needles and the requirements of the case from a few hours to a month.

The most dramatic method of applying radium is by the concentration of several grammes into a "Radium bomb," which is virtually used as a source of intensely hard X-rays, the patient being placed at a distance of a foot or more from the centre of the bomb. In the arrangement devised by Dr. Cheval of Brussels 4 grammes of radium are embedded in the centre of a huge block of lead weighing over a ton and supported on steel girders in the ceiling of one room and the floor of the room above. A double cone cut in the lead allows of the exposure of two patients to intense $\gamma$ ray radiation, one above and one below. So long as the attendants keep outside the cones, which are marked out by circles on the floor and on the ceiling, they are perfectly safe, whilst by an ingenious arrangement the radium itself can retire into a recess in the lead block or advance to the centre of the cone. The method is costly and very extravagant of radiation, but the results are said to be extremely good.

### THE CLINICAL RESULTS OF TREATMENT BY RADIUM.

The results of radium treatment in cases of rodent ulcer and carcinoma of the cervix of the tongue and of the breast are well known, and have been fully described in recent articles. I shall confine myself to describing certain cases of my own in regions where surgery would be difficult or impossible, as I think that one can thus best illustrate the wide range over which radium can be used with success.

### *Carcinoma of the Palate.* (Fig. 3.)

A man aged 55 came to see me last April complaining of a discomfort in the palate of three weeks' duration. At the junction of the hard and soft palate, and extending on to both and on to the alveolar margin, on the right-hand side, was a hard nodular mass of ulcerated fungating carcinoma. There was a large mass of hard glands in the right anterior triangle of the neck. The tumour was destroyed with my steam cautery and the glands were dissected out completely. Healing was perfect in a fortnight, but three weeks later the tumour recurred.

In May I inserted into the base of the tumour six radon seeds, each containing 1·5 millicuries of radon. In fourteen days the growth had disappeared, and was replaced by granulations, in three weeks healing was complete, in six weeks there was no trace even of a scar and he was wearing his toothplate. He remains in perfect health, with no sign of recurrence.

### *Epithelioma of the Lip.* (Fig. 4, *a*, *b*.)

A man aged 65 was sent to me last September with a huge mass of growth involving the whole of the lower lip and

FIG. 4a.

FIG. 4b.

*Epithelioma of Lip.*

FIG. 5a.

FIG. 5b.

*Carcinoma of Thyroid.*

FIG. 6.

*Carcinoma of Œsophagus (from X-ray photograph).*

FIG. 7.
*Carcinoma of Stomach.*

FIG. 8*a*.

FIG. 8*b*.

*Angioma of Meninges.*

extending almost to the chin. It had started a year previously as a small nodule at the left corner of the mouth. Six needles, each 2 centimetres long and containing 2 milligrammes of radium were inserted into the lip along the margin of the growth and were left in place for a week. In three weeks the great bulk of the growth had disappeared, but a week later two small nodules still remaining were treated by the insertion of small radon seeds. A fortnight later the glands were cleared out from both submaxillary triangles, and as the margin of the lip was very ragged, a narrow strip was taken from it. In such an advanced case a permanent cure is too much to expect, but the immediate result was remarkable, and from the cosmetic point of view it was extraordinarily good. A remarkable feature was the reappearance of a lip apparently destroyed by carcinoma, demonstrating conclusively the selective action of the radiation employed. No visible carcinoma remained, and should recurrences appear they can probably be dealt with by similar means.

*Carcinoma of the Thyroid.* (Fig. 5, *a*, *b*.)

A lady aged 70 came to me last September with the history that a small swelling which had always been present in the neck had for five weeks been growing rapidly and was now producing severe respiratory obstruction. There was a hard, nodular swelling involving both lobes of the thyroid, with several hard glands adjacent to it. It was fixed to the trachea and attached to the skin. It was regarded by myself and by Mr. Hayward Pinch as a typical carcinoma of the thyroid. It grew so rapidly that in ten days the circumference of the neck had increased by $1\frac{1}{2}$ inches. Twenty radon seeds, each containing 1·5 millicuries of radon, were inserted through two small punctures, one on each side of the trachea, and were distributed as far as possible throughout the tumour. In ten days the tumour had completely vanished, and although the neck was very thin, the thyroid gland could no longer be felt. The circumference of the neck had diminished by three inches, and all symptoms of obstruction had disappeared. She remains in perfect health.

*Carcinoma of the Œsophagus.* (Fig. 6.)

A man aged 72 had suffered from increasing difficulty in swallowing for three months, and he could now only swallow sips of water with great difficulty. A tall, powerfully-built man, he had lost three stone in weight and he was so weak that he could scarcely stand. An X-ray film showed that the œsophagus was obstructed by a malignant growth ten inches from the teeth. On œsophagoscopy a large ulcerated mass of growth was seen encircling the œsophagus, bulging prominently inwards on the left side. Into the substance of the growth eight radon seeds were inserted, each containing 1·5 millicuries. In twenty-four hours there was a distinct improvement in swallowing, which may, however, have been due to the passage of a bougie at the operation. In a week he was taking ordinary soft foods and was obviously gaining weight and strength. In four weeks he was taking ordinary full diet, with the sole precaution that he should eat slowly. In six weeks he had gained nearly two stone in weight and appeared to be in perfect health.

*Carcinoma of the Stomach.* (Fig. 7.)

A man aged 53 had complained for three months of epigastric pain increased by food, of loss of appetite, and of loss of weight, which he estimated at two stone. On exploration a firm, smooth, œdematous tumour of greyish colour was found involving the lesser curvature and extending upwards almost to the cardiac orifice. It was regarded as a carcinoma of a rapidly growing type, and it was obviously quite inoperable. There were, however, no enlarged glands. Into the substance of the tumour were inserted six platinum needles, each 4 centimetres long and containing 4 milligrammes of radium element. They were so arranged as to radiate from a centre on the anterior surface of the stomach, and the threads collected here were buried in an invagination of the anterior wall of the stomach, formed as for a Witzel's gastrostomy. The threads were brought out in a bunch through the laparotomy opening, which was then closed. Five days later the needles were withdrawn without difficulty. The patient made a normal recovery, his pain ceased, his appetite returned, and in three months he had regained his normal weight and was the picture of health.

*Angioma of the Meninges.* (Figs. 8, *a*, *b*.)

A man aged 45 had suffered for years from fits at long intervals. Recently the fits had become more frequent and severe, and they were now accompanied by loss of consciousness. They were Jacksonian in type, commencing in the left hand and spreading to the face and leg. Beyond a slight increase in the deep reflexes on the left side, there was little to be made

D

out on a neurological examination. On exposing the right Rolandic area by means of an osteoplastic flap formed with my craniotome, and on opening the dura, a mass of huge interlacing veins was found occupying the whole of the upper portion of the field and forming a vascular tumour about three inches in diameter. As no direct treatment seemed practicable, the dura was closed and on to its outer surface were sutured six radium needles, each 2 centimetres long and containing 2 milligrammes of element. The needles were arranged as the spokes of a wheel, and the attached threads were brought out through the central hole in the bone flap. The bone was replaced, the threads brought through the scalp flap, and the scalp was sutured into position.

The after history of the case was somewhat remarkable. Three days later he noticed a weakness of the left arm, which developed next day into a complete paralysis. On the following day the needles were drawn out by threads without difficulty, having been in position for five days. A week later he had completely recovered the use of the arm. There has been no recurrence of the fits, and he seems to be in perfect health.

These cases show the effects which radium can produce in a great variety of situations. They show how wide are its powers, and they suggest that where we have failed it is rather our method of application than radium itself which is at fault. They suggest to me that a new field of surgery is opening up before us, the limits of which it is impossible to foresee.

## Figures and Tables

**Fig. 1. f1:**
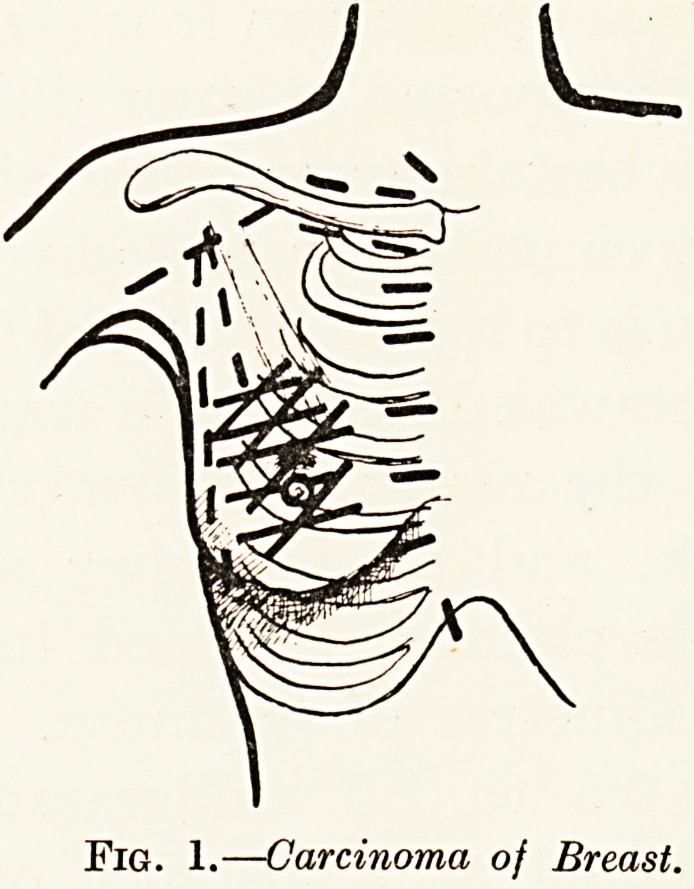


**Fig. 2a. Fig. 2b. f2:**
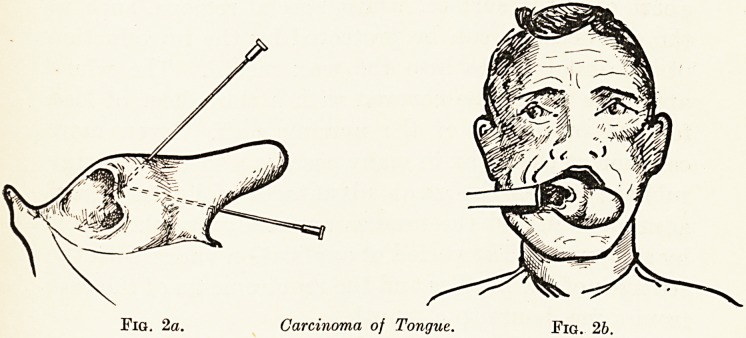


**Fig. 3. f3:**
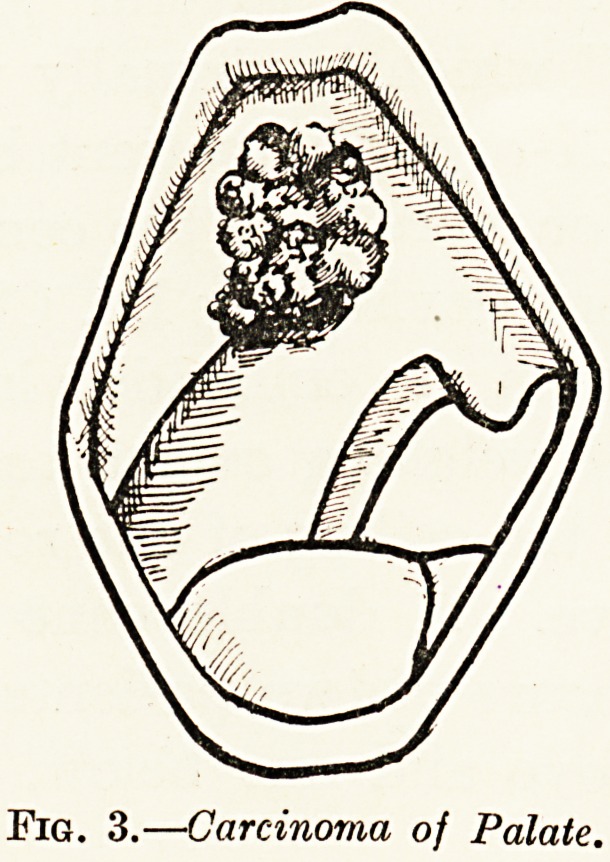


**Fig. 4a. Fig. 4b. f4:**
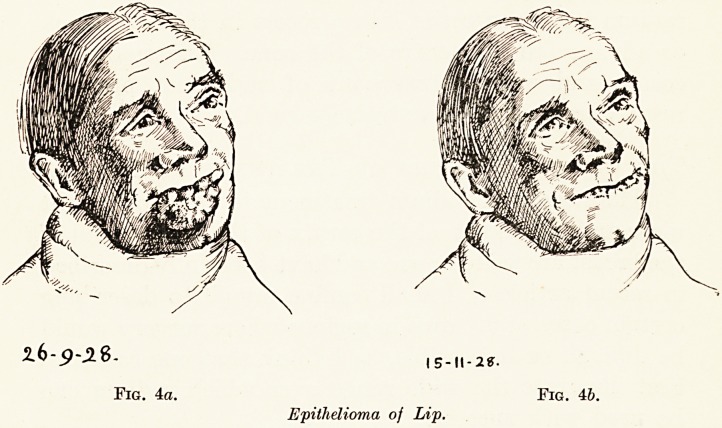


**Fig. 5a. Fig. 5b. f5:**
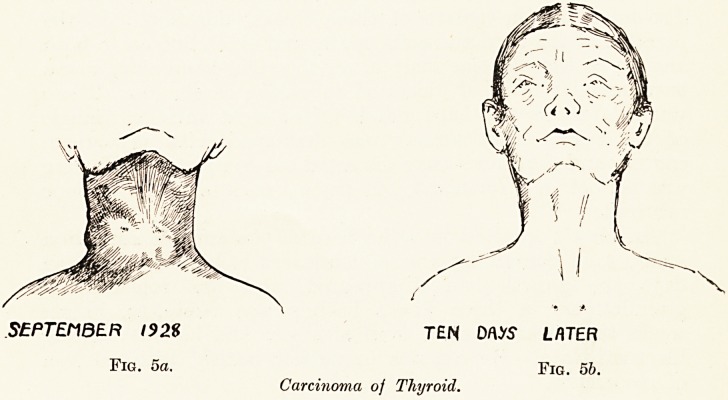


**Fig. 6. f6:**
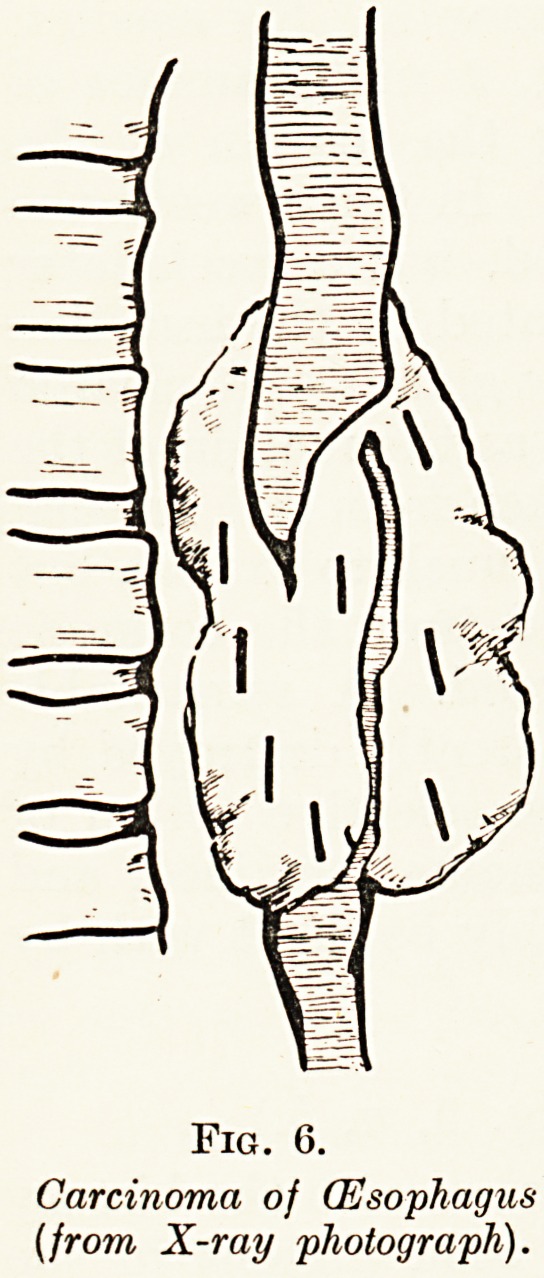


**Fig. 7. f7:**
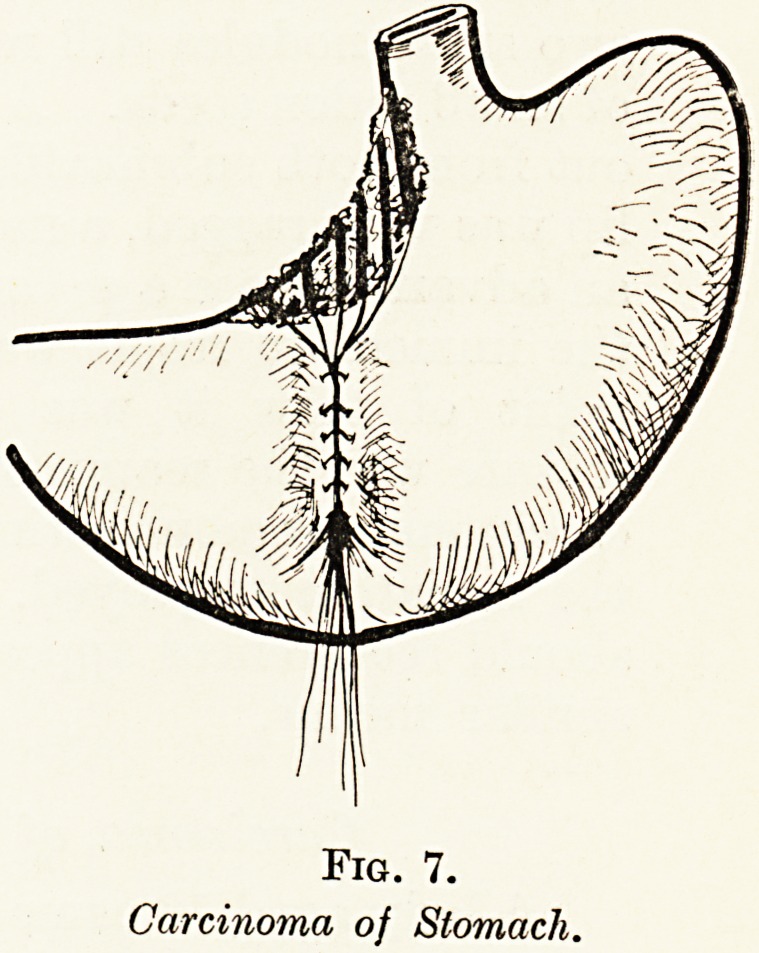


**Fig. 8a. Fig. 8b. f8:**